# A graphene gold nanocomposite-based 5-FU drug and the enhancement of the MCF-7 cell line treatment

**DOI:** 10.1039/c9ra05669f

**Published:** 2019-10-02

**Authors:** Mohamed Fathi Sanad, Ahmed Esmail Shalan, Shereen Magdy Bazid, Esraa Samy Abu Serea, Elhussein M. Hashem, Shimaa Nabih, Md Ariful Ahsan

**Affiliations:** Basic Science Departments, Modern Academy for Engineering and Technology Maadi Egypt; Central Metallurgical Research and Development Institute (CMRDI) P.O. Box 87 Helwan Cairo 11421 Egypt a.shalan133@gmail.com; Departments of Biochemistry, Faculty of Science, Mansoura University Mansoura Egypt; Chemistry & Biochemistry Department, Faculty of Science, Cairo University Cairo Egypt; Chemistry Department, Faculty of Science, Ain-Shams University Abbasia Cairo Egypt; The University of Texas at El Paso 500 W University Ave El Paso TX 79968 USA mfsanad@miners.utep.edu

## Abstract

There is no doubt that cancer is now one of the most formidable diseases in the world; despite all the efforts and research, common treatment routes, including chemotherapy, photodynamic therapy, and photothermal therapy, suffer from different limitations in terms of their efficiency and performance. For this reason, different strategies are being explored to improve the efficiency of the traditional drugs reported to date. In this study, we have redirected the function of one of these drugs (5-fluorouracil, 5-FU) by combining it with a graphene–gold nanocomposite in different molar ratios that has been exceedingly used for biological research development. The high activity of the graphene–gold material enables it to produce reactive oxygen and ions, which display good anticancer and antioxidant activity through the scavenging of the DPPH, SOD and GP_*x*_ radicals; in addition, different characterizations have been used to confirm the structure and morphology of the obtained samples. Highly potent cytotoxicity against the MCF-7 cells was achieved with the drug combination containing the nanocomposite. All the results, including those obtained *via* cytometry, indicate that the combination of 5% graphene–gold nanocomposites with 5-FU exhibits a higher antitumor impact and more drug stability than pure 5-FU.

## Abstract 2

Graphene–gold nanocomposite materials combined with (5-fluorouracil, 5-FU) drug show high potent cytotoxicity against MCF-7 cells, can decrease HER-2 gene expression and cause cell cycle arrest.

## Graphical abstract

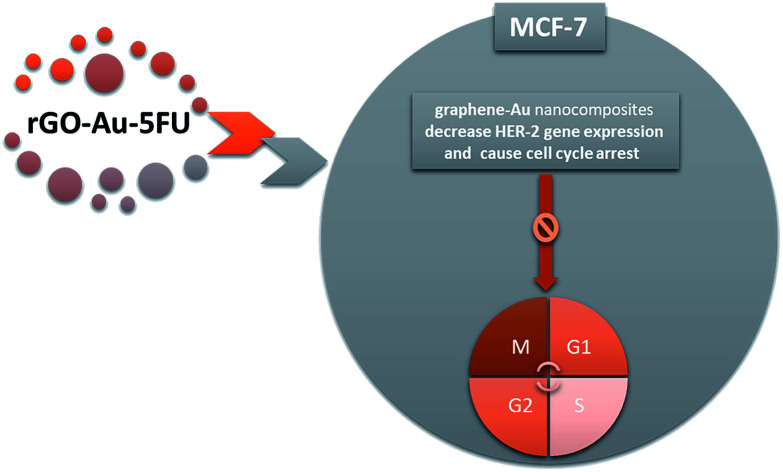

## Introduction

Breast cancer is a formidable disease that is now faced by a large population of women.^1^ Statistical studies indicated that the majority of cases were about 9.7 million in 2002 and are expected to reach 16 million by 2025, especially in developing countries.^2–9^ Many parameters may increase the efficacy of 5-fluorouracil (5-FU) against the development of the disease as breast cancer includes genetic, environmental, natural and physiological factors;^10–14^ chemotherapy, hormone therapy and targeted therapy summarize the main approaches that are approved for curing breast cancer, but each method has its own side effects and drawbacks. Chemotherapy has very dangerous side effects; on the other hand, hormone and targeted therapy are selective to various types of cancers. Nowadays, the knowledge of the 5-FU route of killing MCF-7 has led to novel modifications that have increased the anticancer activity of this drug. Otherwise, the clinical trials of the drug showed that in most cases, the anticancer activity of 5-FU was inhibited due to the resistance of the drug and its low stability. In this study, we have discussed an approach for the new modification of 5-FU, which may act as a large-spectrum anticancer agent by inhibiting very important biological processes, or by being incorporated into genetic molecules and stopping their main functions.^15–19^ In 2008, researchers modified the breast cancer drug to be more operative by coating it with a polymer that had highly biodegradable characteristics against many types of cancer diseases. Sato and his coworkers achieved good results after using oxaliplatin and 5-FU together.^20^ From these previous results, it can be speculated that in the future, the breast cancer treatment strategies will include the incorporation of promising nanoscale composites inside the matrices of drugs.^21^ Gold (Au) nanoparticles have been applied for the treatment of different diseases, including venereal problems, heart disease, cancers, and dysentery, and the diagnosis of some diseases.^22^ About 40 years ago, only few gold-based drugs were applied in preclinical and clinical trials, but different studies were conducted to evaluate the medicinal work. Gold nanoparticles (Au NPs) are considered as a promising anticancer drug due to their unique characteristics such as improved cytotoxicity and easy route of synthesis.^23–27^ The incorporation of Au into cancer cells is considered as a promising treatment method as Au is one of the essential elements for cell cycle progression in the human body.^28–32^

On the other hand, among a wide range of nanocomposites, graphene derivatives have received significant attention and research focus in diverse application areas, including nanomedicine, due to their extraordinary physical and optical characteristics.^33–35^ Intensive research has been applied to investigate the biomedical application of graphene-based nanomaterials as drug-delivery vehicles for cancer treatment, which is considered as a novel therapeutic aspect for performing nanoscale-based chemotherapy coupled with photothermal therapy. Recent development in the functionalization of graphene was achieved through the use of a wide range of materials, including small molecules and biomolecules, to overcome the limitations of reduced graphene oxide (rGO) nano-carriers and thereby make these systems fit for the delivery of treatment agents.^36^ Herein, we used a very simple wet chemical method to make a nanocomposite combination of 5-FU and gold–graphene nanosystems. To obtain the reduced graphene oxide–gold nanocomposites (rGO–Au), graphene was prepared by the modified Hummers' method and doped with gold nanoparticles reduced *in situ* using sodium borohydride. The designed nanosystems were incorporated into the 5-FU drug to enhance both the targeting of the drug and its cytotoxicity against the tumor cells. Different characterization techniques, such as XRD, SEM, TEM and cell cycle analysis, were used to confirm the structure, morphology and realization of the aim of application; furthermore, we obtained an antioxidant report by investigating the DPPH assay, SOD activity and GP_*x*_ enzymes; the cytotoxicity and anticancer activity of the drug against the MCF-7 cells were determined by IC_50_, MTT assay and flow cytometry. All the obtained results fit well with the result stating that graphene–gold nanocomposite-based 5-FU is an economically efficient drug for curing breast cancer.

## Experimental

### Preparation of graphene oxide and graphene–gold nanoparticles

Typically, graphene oxide (GO) was prepared from pure graphite using the modified Hummers' method.^36^ In this method, a mixture of sulfuric and phosphoric acid in a certain ratio (1 : 1, v/v) was prepared and stirred for several minutes. Then, graphite powder was added to the mixture under constant stirring. Potassium permanganate (KMnO_4_, 1 M) as a strong oxidizing agent was added slowly to the solution. This mixture was stirred for 6 h until it attained a dark green color. Excess potassium permanganate was removed using hydrogen peroxide (H_2_O_2_), which was dropped slowly into the mixture followed by stirring for a very short time. After adding hydrochloric acid, the powder was separated and washed correctly. It was then dried using a dryer oven at 85 °C for 2 days to produce the powder of graphene oxide (GO), which was reduced *in situ* to reduced graphene oxide (rGO).^37–39^ In addition, gold (Au) was incorporated inside the GO matrix by *in situ* reduction using sodium borohydride (NaBH_4_, Aldrich) to obtain the desired graphene–gold nanoparticles (rGO–Au).^40^

### Preparation of the graphene–gold–(5-FU) nanocomposite system

The graphene–gold–(5-FU) hybrid nanocomposite was synthesized by pore capping a definite amount of the prepared rGO–Au nanoparticles (0.5 g) with the drug (5-FU, 0.1 g) in 100 mL distilled water, followed by continuous stirring under ambient conditions for 2 hours through a wet mechanical combination as mentioned in our previous study.^41–43^

### Physical characterization

The obtained powders were analyzed *via* the PANalytical MPD diffractometer using Cu Kα1 and Kα2 radiation; X-ray diffraction patterns were clearly detected in the 2 theta diffraction range from 20° to 100°. The diffraction peaks were treated with the riveted refinement pointing using the MAUD program, and any imperfections and defects were monitored using a sample of LaB_6_-based NIST SRM-640b. The morphology of the prepared drug was investigated by a field-emission scanning electron microscope (FESEM) that was connected to a JEOL 6340 electron microscope detector. Furthermore, we calculated the Gaussian-size distribution using the *ImageJ and Normalizing Histogram Origin Program*. Transmission electron microscopy (TEM, JEOL 2100) was used to confirm the nanostructure and graphene–gold cross-interactions.

### Antioxidant activity measurements

For the investigation of the antioxidant activity of the modified drug, we used three types of free radicals: 1,1-diphenyl-2-picrylhydrazyl (DPPH), superoxide dismutase (SOD) and glutathione peroxidase (GP_*x*_); typically, 1 mL of 0.1 mM DPPH methanolic solution was added to 100 μL suspension of all prepared powders, and the reaction was allowed to occur under ambient conditions for a short time of about 30 minutes. Absorbance (*A*) values were obtained using a spectrophotometer with 200 μL of 0.2 mM DPPH as the (−) control and 100 μL of ascorbic acid as the (+) control and by the detection of peaks at (*λ* = 490 nm). The antioxidant% was detected by increasing the inhibition rate, which had already been determined using the following equation:
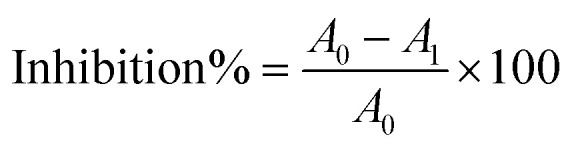
where ‘*A*_0_’ is the control absorbance and ‘*A*_1_’ is the absorbance in the presence of the powders. Superoxide dismutase (SOD) was prepared by mixing 10 mL of 100 mM of phosphate buffer calibrated at pH = 8. GP_*x*_ was also prepared by the same method with the same concentrations. In the case of other samples, the same steps were followed for obtaining the UV-Vis plot and conducting the calculations of the inhibition% with different peak absorption wavelengths.

### Analyzing the viability of the MCF-7 cells

The MCF-7 cells as a model for breast cancer cells were cultured in RPMI supplemented with a medium containing fetal bovine serum (FBS) and some type of antibiotics. The cells were continuously maintained at a temperature of near 35 °C under controlled air and humidity; the culture medium was changed twice weekly. The MTT assay was typically used to detect cell viability while the cells were under treatment. MTT is considered a salt, which is soluble in water and degraded by the action of an enzyme existing in the tumor cells; moreover, it most likely works as a sensor for cell viability. As widely mentioned, the tumor enzyme breaks down the tetrazolium ring into an insoluble byproduct (usually formazan), whose concentration is directly proportional to the number of viable cells. Optical density was plotted *versus* the amount of viable cells, and the IC_50_ value of each drug was measured using the MASTER PLEX software. The percentage cell viability was then calculated with respect to control.

Apoptosis is one of the most important parameters that should be characterized for this kind of research to understand the mechanism of interactions and the biological concept of cell death. It usually proceeds by the translocation of phosphatidylserine from the inner leaflet to the membrane surface and can be assessed using an annexin V antibody. The MCF-7 cells were fixed inside a 6-well plate at a concentration of 2.4 × 10^5^ cells per mL and then incubated for 48 hours until all cell attachment was completed. After this, the seeded cells were treated with the prepared nanocomposite-combined 5-FU samples at the previously monitored IC_50_ concentration value. Incubation was then sustained again for 72 hours; subsequently, about 5 μL of annexin V-fluorescein isothiocyanate (annexin V-FITC)-labeled antibody and 6 μL of propidium iodide were added to produce a distinguishable color to stain the tumor cells; the cells were then placed in a dark place at room temperature for 30 minutes. Afterwards, the stained tumor cells were investigated by flow cytometry.

### RO assay protocol methodology

RO species were determined through the assay kit ab139476 that enabled the real-time monitoring of the reactive oxygen species (ROS) production in the living cells using flow cytometry *via* a microplate reader. The ROS assay protocol is dependent on the fluorescent dyes that detect the oxidative stress reagent (Green, excitation/emission of 490/530 nm) for the detection of the total ROS. To perform this experiment, we supplemented the RO inhibitor into the MCF-7 control cells and kept them for varying time intervals before and after treatment with the composite; after this, we discarded the detection mix, washed the samples and analyzed them with a fluorescence microscope, or analyzed them *via* flow cytometry and/or microplate reader without washing.

## Results and discussion

**Fig. 1:**
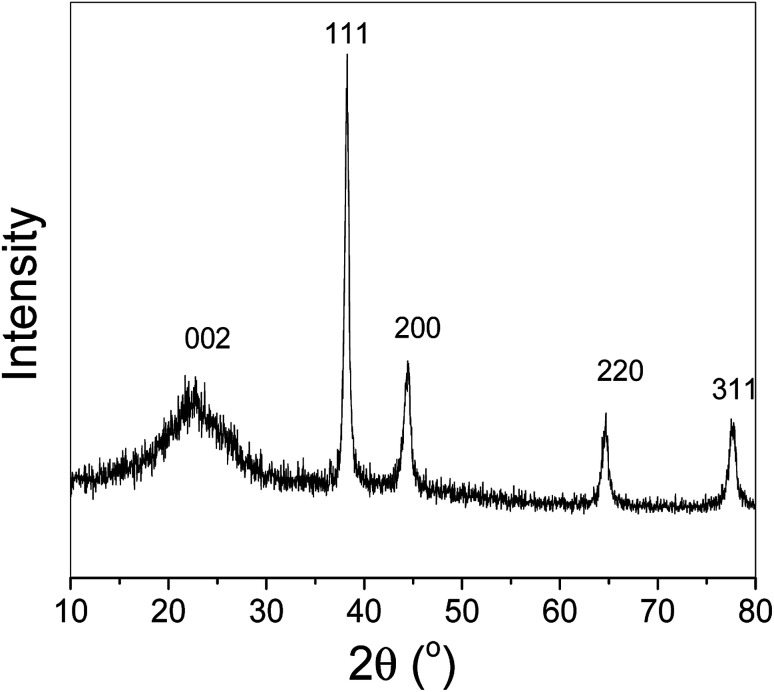
XRD pattern of the prepared nanosystem rGO–Au–5-FU (5%). The prepared sample matched the rGO–Au nanocomposites structure. No other peaks appeared, indicating the purity of the samples.

Fig. 1 shows the XRD pattern of the rGO–Au–5-FU (5%) nanocomposite materials, exhibiting the diffraction lines of both graphene and the gold nanoparticles attached to it without impurities in the prepared sample (Miller indices are clearly provided in the figure). The results indicate that the peak at around 25.0° (0 0 2) corresponds to the diffraction of the reduced graphene structure. Moreover, the position of the (0 0 2) peak can be applied to determine the distance between the attached graphitic layers or d spacing. In the case of our composite materials, this value was 6 nm for rGO–Au–5-FU (5%); the other two peaks at 220 and 311 were related to gold nanoparticles.

**Fig. 2:**
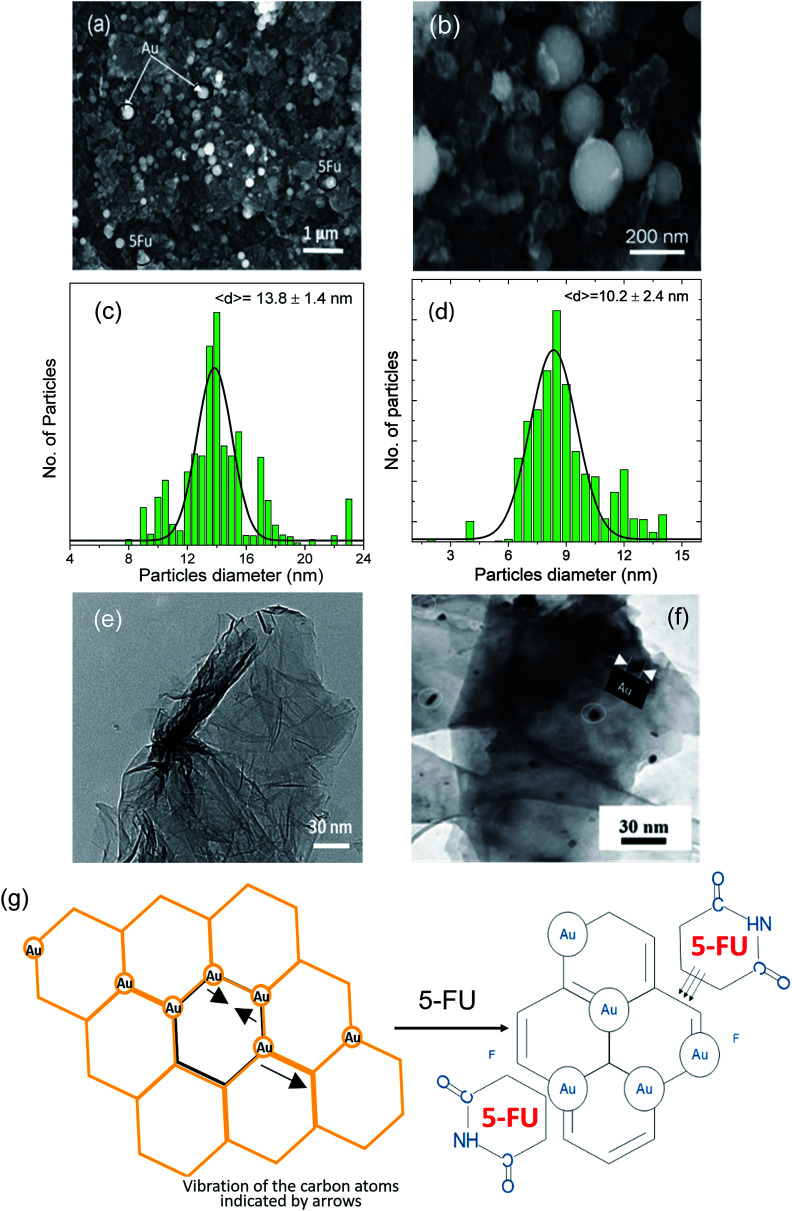
(a and b) FESEM image of the prepared rGO–Au–5-FU (5%) at low and high magnification, respectively. (c and d) Gaussian-size distribution of the obtained rGO–Au–5-FU nanoparticle materials as well as the obtained gold particles inside the rGO–Au nanocomposite. TEM image of (e) the prepared graphene oxide, (f) rGO–Au (5%) and (g) the schematic of the proposed rGO–Au nanocomposite showing the vibration of the carbon atoms indicated by arrows and the combination between the rGO–Au composite and 5-FU structure.^44–46^

Fig. 2 shows the structure of the prepared nanosystem powders. As shown in Fig. 2a, the scanning electron microscopy image indicates a clear combination between different components in a homogeneous network. Furthermore, Fig. 2b shows the formed rGO–Au–5-FU nanocomposites under a high-resolution magnification that illustrates the existence and distribution of both Au and 5-FU particles in the network structure of reduced graphene oxide. We can observe the gold nanoparticles as well as the 5-FU particles attached to the graphene arrays in a symmetrical way. In addition, we calculated the Gaussian-size distribution for the obtained rGO–Au–5-FU materials to detect and confirm the small size and homogeneity of these materials and ensure that the particles were small enough to be easily incorporated into and penetrate the cell membrane. The obtained results affirmed the existence of nanoscale-sized particles with the diameters of 13.8 ± 1.4 nm, as illustrated in Fig. 2c; thus, the obtained rGO–Au–5-FU nanosystem possessed good features towards enhancing the cytotoxicity activity of the drug against breast cancer depending on the effective delivery of drugs by loading 5-FU on the nanocomposites. Subsequently, the clear homogenous size distribution for the pure reduced graphene oxide sheets (Fig. 2e) shows gold particles and 5-FU particles. In addition, the particle size distribution of the Au nanoparticles was calculated in the same way from the obtained SEM results through the ImageJ software and found to be (10.2 ± 2.4) nm, as illustrated in Fig. 2d. To gain a deeper understanding of the obtained rGO–Au–5-FU nanosystem, transmission electron microscopy (TEM) measurements were performed; Fig. 2f clearly shows the incorporation of gold nanoparticles inside the whole matrix of the reduced graphene oxide nanocomposite. In the TEM images, we can see nanoscale spherical nanoparticles, which are distributed in the whole matrix of graphene nanoparticles of around 30 nm thickness. Through this characterization, we can confirm the size, structure and purity of the prepared samples. Fig. 2g shows the schematic of the proposed reduced graphene oxide sheet in the presence of gold nanoparticles to form the rGO–Au nanocomposite, illustrating the vibration of the carbon atoms indicated by arrows. The mechanism for obtaining the proposed structure of the rGO–Au nanocomposites has been reported in the literature.^44–46^ In addition, to probe and investigate the mechanism for attaining the anticipated rGO–Au–5-FU nanocomposites, we speculated the formation mechanism of the rGO–Au–5-FU nanocomposites, as illustrated in Fig. 2g.

**Fig. 3:**
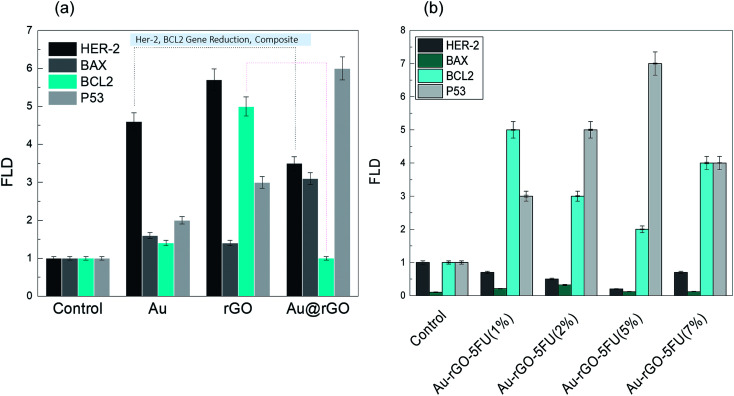
Expression levels of BaX, Her-2, BcL2 and P53 were determined by RT-PCR in MCF7 cells treated with (a) Au, rGO and the Au@rGO nanocomposite, (b) different ratios of Au–rGO–5-FU. All tests were performed in triplicate and the results are reported as the mean ± SEM.

We observed the most potent apoptotic induction when the cells were treated with the rGO–Au–5-FU nanocomposites (5%), and this revealed the addition of the Au nanoparticles into the drug, which increased the anticancer property of the drug. Many studies focus on the treatment systems that arrest the MCF-7 cell cycle at the growth phases and then affect the apoptotic action;^47–52^ hence, the effect of the rGO–Au–5-FU nanocomposites (5%) will determine the mechanism of destruction of the MCF-7 cells. Moreover, to understand the proposed mechanism in this study, we checked the gene expression through RT-PCR detection. The investigation of the gene expression of the MCF-7 cancer cells treated with the rGO–Au–5-FU nanocomposites (5%) *via* RT-PCR recognition for 72 hours indicated lowest values of Bcl-2 and Her-2 in the case of rGO–Au–5-FU nanocomposites (5%) when compared with the cases of other samples (Au, rGO, and Au–rGO through RT-PCR recognition) and the cell control. This also indicated an increase in the expressions of pre-apoptotic genes Bax and P-53, as shown in Fig. 3a. Subsequently, for more confirmation, the same detection was conducted *via* the investigation of the gene expression of MCF-7 cancer cells treated with different ratios of the rGO–Au–5-FU nanocomposites (1, 2, and 7%), as shown in Fig. 3b. The obtained results affirm that the rGO–Au–5-FU nanocomposites (5%) provide the best results as compared to the rest of the samples (Au, rGO, and Au–rGO) and the cell control.

**Fig. 4:**
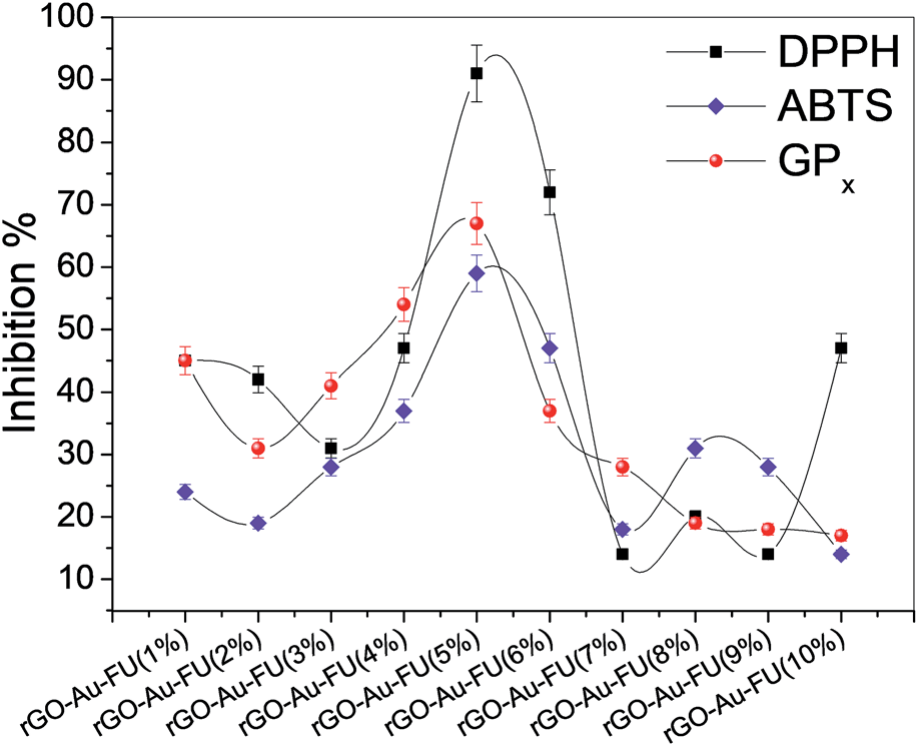
Inhibition% of DPPH, ABTS and GP_*x*_ free radical scavenging activity for all rGO–Au–5-FU fractions. All tests were performed in triplicate and the results are reported as the mean ± SEM.

In addition, the prepared materials used to detect the inhibition activity of different radicals are shown in Fig. 4. The scavenger study results show the inhibition activity of DPPH and the antioxidant activity of the prepared drug. Furthermore, the obtained data confirmed that the antioxidant activities of the samples followed the sequence rGO–Au–5-FU (5%) > rGO–Au–5-FU (1%) > rGO–Au–5-FU (2%) > rGO–Au–5-FU (8%) > 5-FU alone to check the inhibition activity. As discussed in our previous study, the inhibition% of rGO, rGO–Au and 5-FU alone was studied, and the inhibition activity of the radicals of these materials was found to be about 20%, which considered to be very low compared to the different ratios in the current study.^6^ Furthermore, it can be observed that the inhibitory action is increased to the maximum level with 5% and then decreased again by increasing the potent content value, as illustrated clearly in Fig. 4. The results were almost the same for the SOD and GP_*x*_ radical assays, confirming that the nanosystem with the highest percentage of drug had the best results. Therefore, we can conclude from these results that rGO–Au–5-FU (5%) has the highest antioxidant activity.

**Fig. 5:**
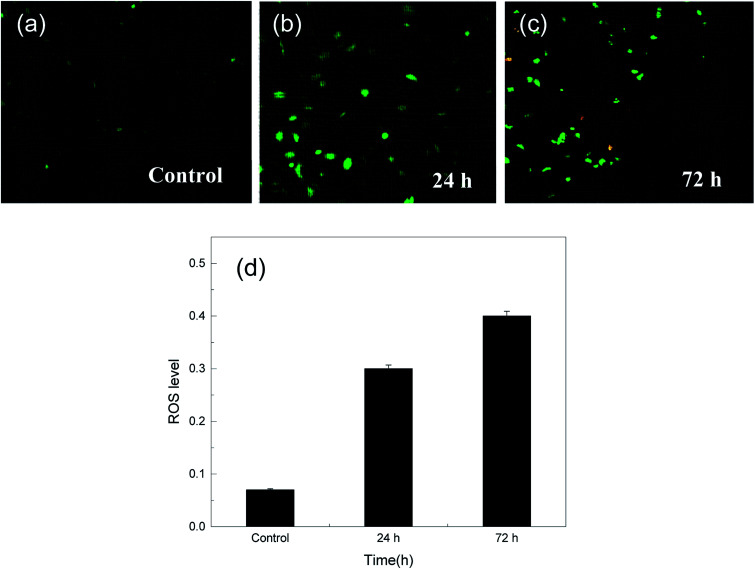
(a) ROS production in the MCF-7 cell line after treatment with rGO–Au–5-FU after (b) 24 and (c) 72 hours of treatment. Cells were incubated for 1 and 3 days in the presence of 20 μM of the prepared sample. (d) Show the relation between ROS levels with time. ROS was detected by staining the cells with the DCFH-DA cellular RO detection assay kit according to the manufacturer's instructions. ROS generation was observed under a fluorescence microscope at 200× magnification.

In addition, the reactive oxygen species or radicals (ROS) play an essential role in different cellular processes and are known to be vital for cell proliferation at basal levels.^53,54^ However, RO can become cytotoxic to cells at high concentrations, an effect that related to RO is frequently dedicated for different therapeutic applications.^54^ There are different types of methods for the detection of free radical production in cells; the application of 2′,7′-dichlorodihydrofluorescein diacetate (DCFH-DA) is one of the most commonly used systems for directly measuring the redox state of a cell;^55^ this involves breaking of the two-ester bonds in its structure, which in turn produces H_2_DCF, which is oxidized by the accumulation of RO species in the cell and turns into fluorescent DCF. Therefore, we can monitor this by the increase in fluorescence at 530 nm when the cell is excited at 490 nm. Fig. 5(a–d) show ROS detection over the MCF-7 cells after treatment with the drug for one day and 3 days. The results show that a level of about 0.5 after 3 days is considered a very good ratio as compared to that in other studies, which exceeds an ROS level of 10; although this level is not sufficient to break the genetic materials in cells, *via* the cytotoxicity results, it can be understood that it causes necrosis and apoptotic death.^56–58^

**Fig. 6:**
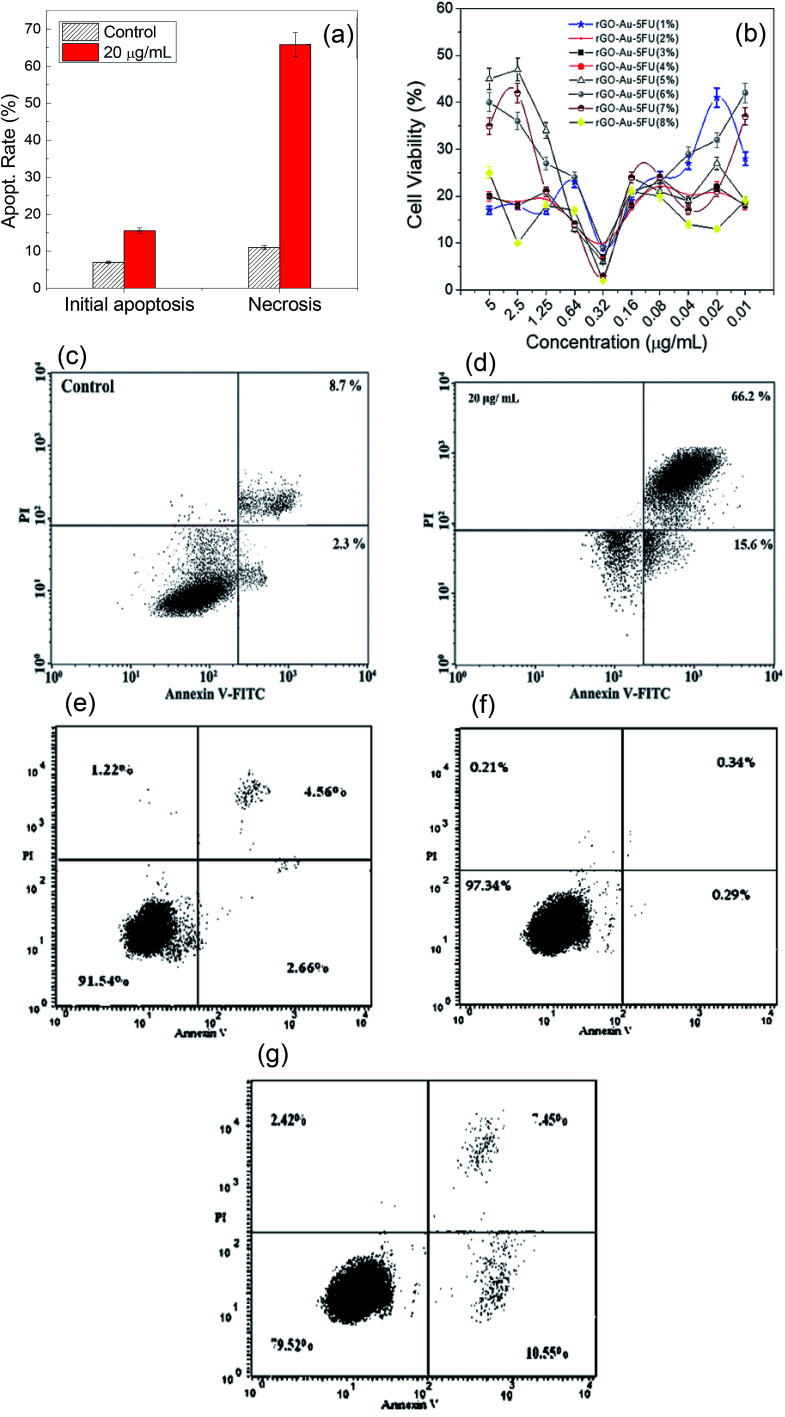
(a) The effect of the rGO–Au–5-FU prepared samples on the apoptosis of the MCF-7 cells, which were incubated for 72 hours. (b) Cell viability of different ratios of Au–rGO–5-FU. (c and d) The effect of rGO–Au–5-FU and (e–g) the effect of Au, rGO and Au–rGO prepared samples on the MCF-7 cell apoptosis. Cells were incubated for 72 hours. Viable and dead cells were observed by the annexin V-FITC flow cytometry method. All tests were performed in triplicate, and the results are reported as the mean ± SEM.

In addition, Fig. 6(a and b) show the effect of the rGO–Au–5-FU nanosystem samples on the viability of the MCF-7 cells cultured in six-well plates for one day and represent a comparison of the cytotoxic effect between all samples with different concentrations as well as pure 5-FU after 72 hours of treatment. The results indicated that rGO–Au–5-FU (5%) was significantly more cytotoxic than the other samples at lower concentrations. All types of cell cycle phases were studied using DNA flow cytometry analysis. This nanosystem has the ability to penetrate the cell membrane that converts liquids and enzymes into different active metabolites such as fluorodeoxyuridine monophosphate (FdUMP) and fluorouridine triphosphate (FUTP). Fig. 6(c and d) show the apoptosis study results for the MCF-7 breast cell lines treated by rGO–Au–5-FU (5%) nanocomposites obtained using a flow cytometry instrument (annexin V-FITC), which show an acute reduction in the number of living cells caused by this mechanism; this indicates that the initial apoptosis has been increased by about 60% and the late apoptosis has been increased by 40%. This provides a good indication of the effect of the nanocomposite modification as compared to the obtained results for pure 5-FU. Usually, it increased the apoptotic death by about 15–35%. Moreover, Fig. 6(e–g) show the apoptosis induction results obtained using gold, reduced graphene oxide, and gold and reduced graphene oxide together with the prepared nanocomposite-treated cells. We can clearly see that these materials had no observed effect on the breast cells. However, only in the case of the composite, the apoptotic rate increased to 10.5% as compared to the control value of 2.5%, as shown in the obtained results. From the apoptosis results, the proposed mechanism of action of the drug-based nanosystems is that after incorporation of the drug inside the tumor cells, the apoptotic induction is increased by a large extent, especially at the initial stage. It is most likely that the drug enhances some genes that are responsible for cell growth. In addition, the radical scavenging assay shows that the antioxidant activity of the new system is greater than that of pure 5-FU. The inhibition percentage was also improved by increasing the amount of the nanomaterial incorporated to a ratio of about 5% and a concentration of about 20 μg mL^−1^. At this concentration, the surface of 5-FU was completely filled with rGO–Au–5-FU (5%) and the antioxidant activity began to decrease, revealing the decrease in the free surface area of 5-FU. Annexin V-FITC apoptotic induction reached its highest induction value using rGO–Au–5-FU (5%) through arrest in the G2/M phase and reduction in the G1 phase.

## Conclusion

Herein, a novel route was applied to modify the cytotoxicity and targeting technique of the 5-fluorouracil drug by incorporating graphene gold nanocomposites inside the cross-linked matrix of the 5-FU core, as shown in this study. This nanosystem has the ability to penetrate the cell membrane, which converts liquids and then enzymes into different active metabolites such as fluorodeoxyuridine monophosphate (FdUMP) and fluorouridine triphosphate (FUTP). These active biomaterials affect the genetic material synthesis of the breast cancer cells. It was clearly observed that the rate of reduction of the tumor cells was increased to a large extent at the optimal concentration of the nanocomposite. The free-radical scavenging assay also showed that the antioxidant activity of the new system was greater than that of pure 5-FU. In addition, the inhibition percentage was improved by increasing the amount of nanomaterial incorporated up to a ratio of about 5% and concentration of about 20 μg mL^−1^, where the surface of 5-FU was completely filled with rGO–Au–5-FU (5%). At this concentration, the antioxidant activity began to decrease, and this revealed the decrease in the free surface area of 5-FU. The annexin V-FITC apoptotic induction reached its highest induction value with rGO–Au–5-FU (5%) through arrest in the G2/M phase and reduction in the G1 phase. Thus, our new drug based on modified 5-FU for the treatment of breast cancer cells may be a good candidate for preclinical and subsequent clinical studies.

## Conflicts of interest

No contending financial interest is proclaimed by the authors.

## Supplementary Material
